# Catabolism of coniferyl aldehyde, ferulic acid and *p*-coumaric acid by *Saccharomyces cerevisiae* yields less toxic products

**DOI:** 10.1186/s12934-015-0338-x

**Published:** 2015-09-21

**Authors:** Peter Temitope Adeboye, Maurizio Bettiga, Fredrik Aldaeus, Per Tomas Larsson, Lisbeth Olsson

**Affiliations:** Department of Biology and Biological Engineering, Industrial Biotechnology, Chalmers University of Technology, 412 96 Gothenburg, Sweden; Innventia AB, Drottning Kristinas väg 61, 114 28 Stockholm, Sweden

**Keywords:** Biorefinery, Phenolic compounds, Conversion, Coniferyl aldehyde, Ferulic acid, *p*-Coumaric acid

## Abstract

**Background:**

Lignocellulosic substrates and pulping process streams are of increasing relevance to biorefineries for second generation biofuels and biochemical production. They are known to be rich in sugars and inhibitors such as phenolic compounds, organic acids and furaldehydes. Phenolic compounds are a group of aromatic compounds known to be inhibitory to fermentative organisms. It is known that inhibition of *Sacchromycescerevisiae* varies among phenolic compounds and the yeast is capable of in situ catabolic conversion and metabolism of some phenolic compounds. In an approach to engineer a *S. cerevisiae* strain with higher tolerance to phenolic inhibitors, we selectively investigated the metabolic conversion and physiological effects of coniferyl aldehyde, ferulic acid, and p-coumaric acid in *Saccharomyces cerevisiae*. Aerobic batch cultivations were separately performed with each of the three phenolic compounds. Conversion of each of the phenolic compounds was observed on time-based qualitative analysis of the culture broth to monitor various intermediate and final metabolites.

**Result:**

Coniferyl aldehyde was rapidly converted within the first 24 h, while ferulic acid and *p*-coumaric acid were more slowly converted over a period of 72 h. The conversion of the three phenolic compounds was observed to involved several transient intermediates that were concurrently formed and converted to other phenolic products. Although there were several conversion products formed from coniferyl aldehyde, ferulic acid and *p*-coumaric acid, the conversion products profile from the three compounds were similar. On the physiology of *Saccharomyces cerevisiae*, the maximum specific growth rates of the yeast was not affected in the presence of coniferyl aldehyde or ferulic acid, but it was significantly reduced in the presence of *p*-coumaric acid. The biomass yields on glucose were reduced to 73 and 54 % of the control in the presence of coniferyl aldehyde and ferulic acid, respectively, biomass yield increased to 127 % of the control in the presence of *p*-coumaric acid. Coniferyl aldehyde, ferulic acid and *p*-coumaric acid and their conversion products were screened for inhibition, the conversion products were less inhibitory than coniferyl aldehyde, ferulic acid and *p*-coumaric acid, indicating that the conversion of the three compounds by *Saccharomyces cerevisiae* was also a detoxification process.

**Conclusion:**

We conclude that the conversion of coniferyl aldehyde, ferulic acid and p-coumaric acid into less inhibitory compounds is a form of stress response and a detoxification process. We hypothesize that all phenolic compounds are converted by *Saccharomyces cerevisiae* using the same metabolic process. We suggest that the enhancement of the ability of *S. cerevisiae* to convert toxic phenolic compounds into less inhibitory compounds is a potent route to developing a *S. cerevisiae* with superior tolerance to phenolic compounds.

## Background

Lignocellulosic substrates are increasingly gaining attention as raw materials for biofuels and chemicals although numerous challenges on fermentability confront their usage as production platforms [1, 2]. Lignocellulosic substrates are primarily composed of cellulose, hemicellulose and lignin [3]. To disintegrate and make lignocellulosic biomass structurally accessible to enzymatic hydrolysis before fermentation, it is first subjected to a pre-treatment process [4, 5].

Also, the concept of chemical and fuel production in an integrated biorefinery is driving the interest in pulping process streams which are often rich in derivatives of lignin and hemicellulose [6, 7].

Pulping is a well-established technology for biomass disintegration and fractionation to make wood pulps [8]. Chemical pulping is a widespread process, the four classical methods principally used in chemical pulping are the kraft, sulfite, soda, and neutral sulfite semi-chemical pulping (NSSC) processes [9]. Pulping involves cooking wood biomass to obtain cellulose fibers during which delignification takes place and monomeric sugars from the hemicellulose fraction are released into the cooking liquor [10], the cooking liquor is then released as the process streams. Cooking liquor such as spent sulfite liquor, black liquor, delignification stream and pulp residues are useful energy and lignin sources, as well as having potentials for several purposes, including being used for bioethanol and chemical production [11]. In biofuel production, the acids and phenolic compounds derivatives of hemicellulose and lignin released into the process streams act as potent inhibitors against fermenting organisms [4, 12]. In the case of biochemical production, it has been shown that phenolic inhibitors in black liquor can be converted into value added chemicals [13].

The diverse nature of phenolic compounds present a significant challenge, they are thus the least studied and understood of all of inhibitors present in lignocellulosic materials [14]. Although studies have shown that various phenolic compounds such as ferulic acid and coniferyl aldehyde influence specific processes in *S. cerevisiae* [15, 16], the way the yeast cells respond and adapt to various phenolic compounds has not been well investigated. The ability of *S. cerevisiae* to convert particular phenolic compounds under fermentation, such as converting ferulic acid to 4-vinylguaiacol and coniferyl aldehyde to coniferyl alcohol, has been previously reported. Some *S. cerevisiae* strains with increased tolerance to the inhibitory activities of phenolic compounds were also engineered [17, 18]. However, several processes and mechanisms involved in the conversion of phenolic compounds in *S. cerevisiae* remain poorly understood. Information on the possible conversion pathway as well as a comprehensive list of products formed from the conversion is lacking. Apart from the importance of understanding the metabolic process involved with phenolic compound conversion, it is also important to investigate if the conversion products are more, equally, or less inhibitory in comparison with the parent compound. A conversion process that leads to less inhibitory compounds is one of the keys that could be explored for metabolic engineering strategies to develop a more phenolic tolerant *S. cerevisiae*. We have previously observed that inhibitory capacity of phenolic compounds against *S. cerevisiae* is compound specific, we also observed variation in the physiological influence on of phenolic compounds on *S. cerevisiae* [19].

In a lignocellulosic substrate, the different inhibitory compounds work in synergy and limit the chances to assign specific cell physiological response observed (effects) to the compounds inducing such a response. Although, the ability of *S. cerevisiae* to convert some phenolic, such as cinnamic acids have been previously reported [17, 20, 21], the complexity of lignocellulosic substrates and pulping streams makes it incredibly difficult to assign conversion products to specific starting compound during the bioconversion process. Therefore, monitoring the intermediates and products of catabolic conversion and investigating cell response to individual compounds may be best done by studying the effects of the phenolic compounds in a single substrate study. Based on this, we have done a selective study on the interaction of *S. cerevisiae* with three phenolic compounds coniferyl aldehyde, ferulic acid and p-coumaric acid under single substrate cultivation conditions in which only one of the three compounds is present in a cultivation set up.

**Fig. 1:**
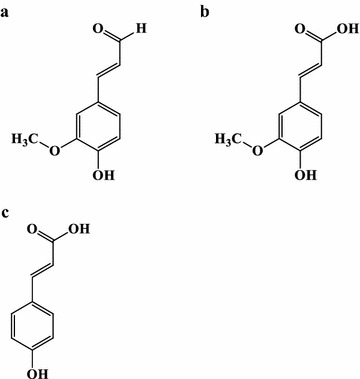
Structures of; **a** coniferyl aldehyde, **b** ferulic acid and **c** *p*-coumaric acid

In the present study, we closely investigated the interactions between yeast and phenolic compounds in a controlled environment, in order to understand the mechanisms and metabolic processes in *S. cerevisiae* which facilitate the conversion of, and resistance to, phenolic compounds. We have studied the conversion of phenolic compounds in order to provide information which is valuable for metabolic engineering and the development of yeast strains with improved tolerance to phenolic compounds. In addition, our investigation intends to pave the way to future research investigating the use of yeast as a catalyst for the potential aerobic conversion of phenolic compounds to chemicals of interest. In this paper, we present results detailing the individual metabolic conversion of three phenolic compounds by *S. cerevisiae*: coniferyl aldehyde, ferulic acid, and *p*-coumaric acid (Fig. 1). The results suggest that there is a previously unreported route that starts with phenolic aldehydes and leads to phenolic alcohols.

## Results

### Effects of coniferyl aldehyde, ferulic acid, and *p*-coumaric acid on cell growth

**Fig. 2:**
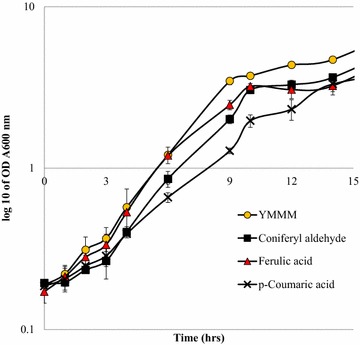
Representative growth curve for aerobic batch cultivation of *S. cerevisiae Ethanol Red* in yeast minimal mineral medium (YMMM), coniferyl aldehyde, ferulic acid and *p*-coumaric acid

We have previously defined the toxicity limits of coniferyl aldehyde, ferulic acid, and p-coumaric acid on *S. cerevisiae* as 1.4, 1.8 and 9.7, respectively using high-throughput microtiter plate growth experiments [19]. The toxicity limits of the different phenolic compounds were defined as the concentration at which the cell performance is reduced by 80 % with respect to the control, and are based on the aspect of the yeast cultivations which were most affected (maximum specific growth rates, or final OD, or prolongation of the lag phase) [19]. In fermentor cultivations, it was found that the yeast cells did not grow in the presence of 1.4 mM coniferyl aldehyde. We therefore reduced the concentration of coniferyl aldehyde used in the cultivations by one concentration step to 1.1 mM in order to successfully cultivate the yeast cells in the presence of coniferyl aldehyde. To study the influence of coniferyl aldehyde, ferulic acid, and *p*-coumaric acid on *S. cerevisiae,* three cultivation experiments were set up. The first cultivation set up was with 1.1 mM coniferyl aldehyde in mineral medium, the second cultivation was with 1.8 mM ferulic acid while the third was with 9.7 mM *p*-coumaric acid. At these concentrations, the compounds did not arrest the growth of *S. cerevisiae*. The yeast grew at different specific rates in the presence of the different phenolic compounds, with the fastest growth being recorded in the presence of coniferyl aldehyde, closely followed by growth in the presence of ferulic acid. The slowest growth was observed in cultivations containing *p*-coumaric acid (Fig. 2). The maximum specific growth rates of the yeast under the influence of coniferyl aldehyde was 0.41 ± 0.07 h^−1^ while it was 0.35 ± 0.02 h^−1^ in ferulic acid. These were not significantly different from the specific growth rate of the control at 0.37 ± 0.02 h^−1^. However, the maximum specific growth rate of the cells in the presence of *p*-coumaric acid was statistically different, and was reduced to 0.29 ± 0.02 h^−1^. In the toxicity ranking carried out in the Bioscreen experiments [19], we observed that the maximum specific growth rates of *S. cerevisiae* were reduced by 80 % in comparison with the control cultivation when each of the phenolic compounds was present, we also observed a prolongation of the lag phase in the presence of coniferyl aldehyde. These were not observed in the bioreactor cultivation. We have attributed the changes in maximum specific growth rate and growth pattern to the scaling up of the experiment from the Bioscreen to bioreactors, which offer a different, better controlled cultivation condition. In the case of a pH related toxicity, which may well be among phenolic compounds, it is very probable that the differences in growth pattern between bioreactor cultivations and Bioscreen cultivations is pH related. The pH of all growth media was set to 5.0 at the start of each cultivation, however, the pH is not controlled in the bioscreen and reduces with time while in the bioreactor cultivations the pH was maintained at 5 throughout the cultivation.

### Effects of coniferyl aldehyde, ferulic acid, and *p*-coumaric acid on the titres and yields of fermentation products

During aerobic growth in batch cultures, *S. cerevisiae* induces aerobic fermentation during which, in addition to biomass, ethanol, glycerol and acetate are produced.

**Fig. 3:**
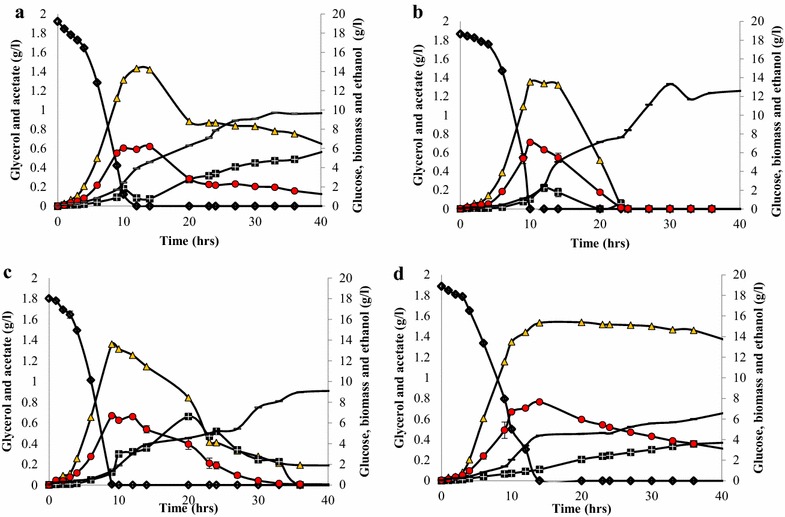
Representative time course metabolite profiles of *Saccharomyces cerevisiae Ethanol Red* in **a** YMMM, **b** coniferyl aldehyde, **c** ferulic acid, **d** *p*-coumaric acid. () glycerol, () acetate, () ethanol, () biomass, () Glucose

Biomass titres were 13.44 ± 0.06 g/l, 9.41 ± 0.05 g/l, 8.19 ± 0.02 g/l and 10.21 ± 0.03 g/l in cultivations containing coniferyl aldehyde, ferulic acid, *p*-coumaric acid and the YMMM control, respectively (Fig. 3). The biomass yields on glucose were 0.08 ± 0.009 g/g, 0.06 ± 0.008 g/g, 0.14 ± 0.07 g/g and 0.11 ± 0.019 g/g in cultures with coniferyl aldehyde, ferulic acid, *p*-coumaric acid and the YMMM control, respectively (Table 1).

**Table 1:** Metabolite profile of *S. cerevisiae* in control, phenolics-free yeast minimal mineral medium control medium in comparison with *S. cerevisiae* presence of each of 1.1 mM coniferyl aldehyde, 1.8 mM ferulic acid and 9.7 mM *p*-coumaric acid

	Titre at the end of cultivation (g/l)	Titre at the end of respirofermentative phase (g/l)	Yield (g/g)	µmax (h^−1^)	Respirofermentative	Respiratory
					q (gg^−1^ h^−1^)	q (gg^−1^ h^−1^)
Yeast minimal mineral medium						
Glucose		0.01 ± 0.005	1	0.37 ± 0.02	3.94 ± 0.04	
Ethanol		6.87 ± 0.1	0.39 ± 0.011		1.53 ± 0.02	
Biomass	10.24 ± 0.03	2.13 ± 0.07	0.11 ± 0.019		0.37 ± 0.02	0.08 ± 0.001
Glycerol	0.06 ± 0.06	1.52 ± 0.1	0.08 ± 0.006		0.30 ± 0.05	
Acetate		0.37 ± 0.02	0.01 ± 0.001		0.03 ± 0.005	
CO_2_	15.18 ± 0.03	2.99 ± 0.1	0.30 ± 0.012		1.56 ± 0.12	
Coniferyl aldehyde						
Glucose		0.04 ± 0.005	1	0.41 ± 0.07	4.68 ± 0.10	
Ethanol		5.73 ± 0.06	0.40 ± 0.01		1.87 ± 0.10	
Biomass	13.44 ± 0.06	1.39 ± 0.03	0.08 ± 0.009		0.35 ± 0.01	0.09 ± 0.005
Glycerol		1.18 ± 0.02	0.08 ± 0.006		0.37 ± 0.04	
Acetate		0.09 ± 0.04	0.01 ± 0.001		0.03 ± 0.002	
CO_2_	16.25 ± 0.07	4.72 ± 0.03	0.34 ± 0.005		1.59 ± 0.12	
Ferulic acid						
Glucose		0.01 ± 0.005	1	0.35 ± 0.02	6.82 ± 0.08	
Ethanol	0.08 ± 0.02	6.57 ± 0.001	0.36 ± 0.005		2.44 ± 0.02	
Biomass	9.41 ± 0.05	1.35 ± 0.014	0.06 ± 0.008		0.41 ± 0.04	0.11 ± 0.006
Glycerol	0.18 ± 0.00	1.35 ± 0.05	0.08 ± 0.002		0.51 ± 0.04	
Acetate		0.12 ± 0.006	0.01 ± 0.001		0.05 ± 0.002	
CO_2_	20.05 ± 0.1	3.34 ± 0.006	0.29 ± 0.01		2.29 ± 0.1	
*p*-Coumaric acid						
Glucose		0.02 ± 0.02	1	0.29 ± 0.02	2.95 ± 0.07	
Ethanol	0.02 ± 0.02	5.4 ± 0.05	0.37 ± 0.011		1.11 ± 0.05	
Biomass	8.19 ± 0.02	1.93 ± 0.05	0.14 ± 0.07		0.29 ± 0.02	0.09 ± 0.003
Glycerol	0.07 ± 0.03	1.32 ± 0.02	0.12 ± 0.002		0.31 ± 0.04	
Acetate		0.08 ± 0.01	0.01 ± 0.001		0.02 ± 0.008	
CO_2_	12.23 ± 0.13	1.54 ± 0.0	0.03 ± 0.005		0.07 ± 0.004	

As enumerated in Table 1, the ethanol yield was highest at 0.4 ± 0.01 g/g in cultures containing coniferyl aldehyde, while ethanol yields were 0.36 ± 0.005 g/g, 0.37 ± 0.011 g/g and 0.39 ± 0.011 g/g in cultures containing ferulic acid, *p*-coumaric acid and the YMMM control, respectively.

The glycerol yields were 0.08 ± 0.006 g/g in cultures with coniferyl aldehyde; 0.08 ± 0.002 g/g with ferulic acid; 0.12 ± 0.002 g/g with *p*-coumaric acid; and 0.08 ± 0.006 g/g in the YMMM control cultivation. The glycerol yield in *p*-coumaric acid was significantly higher than in other cultivations.

After the diauxic shift, at which point all the glucose has been consumed, ethanol, glycerol and acetate start to be assimilated. Assimilation of ethanol, glycerol and acetate was slowed in *p*-coumaric acid cultivations, the metabolites were still present after 73 h of cultivation, whereas they were assimilated within 50 h of cultivation in coniferyl aldehyde, ferulic acid, and in the control cultivations.

### Conversion of phenolic compounds

Interestingly, we observed complete conversion of coniferyl aldehyde, ferulic acid and p-coumaric acid into other phenolic compounds. Conversion of the phenolic compounds was monitored through sampling and analysis of the culture broth at regular intervals during the course of the cultivations. Conversion of coniferyl aldehyde and ferulic acid was initiated by the cells within the first 2 h of cultivation, while the conversion of *p*-coumaric acid was first observed much later. After 24 h all the coniferyl aldehyde had been converted, while ferulic acid and *p*-coumaric acid required a period of over 72 h for complete conversion (Table 2). We observed the concurrent formation of several intermediates during the conversion. Some intermediates such as homovanillin, 2′,5′-dihydroxyacetophenone, coumaran and 3-vanilpropanol from coniferyl aldehyde were very transient, and were only present in the culture broth for a period of about 24 h, whereas other intermediate products, such as 4-vinylguaiacol from both coniferyl aldehyde and ferulic acid, as well as the ferulic acid intermediate from coniferyl aldehyde, were slowly converted into other products over a longer time period (Table 2).

**Table 2:** The conversion products profile of 1.1 mM coniferyl aldehyde, 1.8 mM ferulic acid and 9.7 mM *p*-coumaric acid with time

			0 h	2 h	24 h	48 h	72 h
Coniferyl aldehyde							
Coniferyl aldehyde			+	+	+		
Ferulic acid				+	+	+	
Ferulic acid, isomer				+	+	+	+
Dihydroferulic acid					+	+	+
Homovanillin					+		
2′,5′-Dihydroxyacetophenone					+		
Coumaran					+	+	
3-Vanilpropanol					+	+	
4-Hydroxyphenylethylethanol					+	+	+
Phenyl ethyl alcohol					+	+	+
4-Hydroxyphenylethanol					+	+	+
Benzoic acid, 3-methoxy-4-hydroxy					+	+	+
*p*-Coumaric acid					+	+	+
Benzenepropanoic acid					+	+	+
4-Vinylguaiacol					+	+	+
Benzeneacetic acid							+
Ferulic acid							
Ferulic acid			+	+	+	+	+
Ferulic acid, isomer				+	+		
Dihydroferulic acid				+	+		
2′,5′-Dihydroxyacetophenone					+		
5-Allyl-1-methoxy-2,3-dihydroxybenzene					+	+	
4-Hydroxyphenylethanol					+	+	+
Benzeneacetic acid					+	+	+
4-Vinylguaiacol					+	+	+
Phenylethyl alcohol					+	+	+
*p*-Coumaric acid							
*p*-Coumaric acid			+	+	+	+	+
Coumaran					+	+	+
4-Hydroxyphenylethylethanol					+	+	+
Phenyl ethyl alcohol					+	+	+
2,6-(1,1-Dimethylethyl)phenol					+	+	+

“+” connotes the presence of a compound while a blank space means the compound was absent

**Fig. 4:**
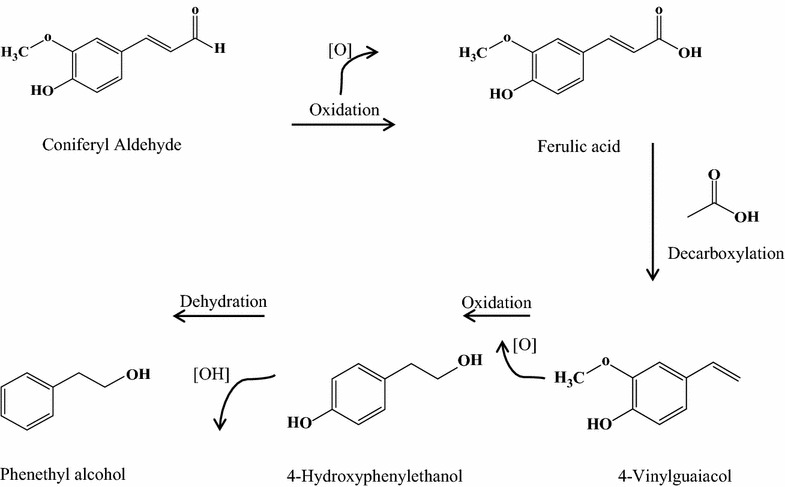
A suggested conversion pattern in the detoxification of coniferyl aldehyde to phenyl ethyl alcohol, based on extracellular metabolites identified in the time evolution data presented in Table 2

During the first 2 h of cultivation, coniferyl aldehyde was initially converted to ferulic acid and ferulic acid isomer, before being further converted to other phenolic acids and other classes of compounds. Ferulic acid was also converted to ferulic acid isomer and dihydroferulic acid during the first 2 h of cultivation, before other conversion products were detected. The conversion trend in *p*-coumaric acid cultivations appeared to have fewer intermediates and products than in cultivations with coniferyl aldehyde and ferulic acid (Table 2). From the time evolution of the conversion products, it is evident that the observed conversion process was a sequential process involving several chemical reactions (Fig. 4). From the observed overlapping of products (Table 2), it is deducible that the chemical reactions involved in the conversion were simultaneously taking place.

### Comparison of inhibition between coniferyl aldehyde, ferulic acid and *p*-coumaric acid their conversion products

**Fig. 5:**
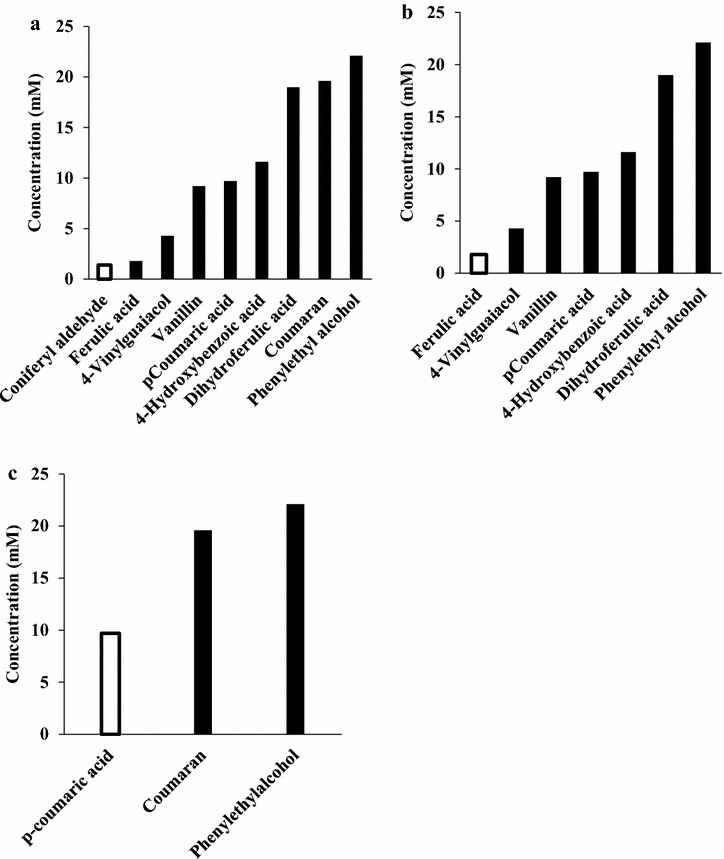
Comparison of toxicity between conversion products (*filled bars*) and their corresponding parent phenolic compounds (*empty bars*): **a** coniferyl aldehyde and its products **b** ferulic acid and its products **c** *p*-coumaric acid and its products

To verify that the conversion of coniferyl aldehyde, ferulic acid and *p*-coumaric acid is a detoxification process, toxicity screening of several conversion products of each of the compounds was carried out and compared to that of their parent compounds. In the toxicity screening, the concentration at which each phenolic compound completely inhibits cell growth was determined similarly to what we had earlier reported [19]. We found that the conversion phenolic products were all less toxic than their parent compounds (Fig. 5). With conversion products such as phenyl ethyl alcohol, the toxicity limits were not reached. The experiment was terminated because of inaccuracy in the OD measurement caused by the strong interference from the colour of the compounds as well as the particulate background resulting from insolubility at higher concentrations. Phenyl ethyl alcohol did not inhibit yeast growth at 22.1 mM as effectively as 1.1 mM coniferyl aldehyde or 1.8 mM ferulic acid. Significantly higher concentrations of other conversion products such as vanillin, dihydroferulic acid, and coumaran, were also needed to inhibit yeast growth to a comparable extent to the coniferyl aldehyde, ferulic acid, and *p*-coumaric acid from which they were derived. This proves that the conversion products were much less toxic than their parent compounds and, therefore, the conversion serves as a detoxification process.

## Discussion

Our results indicate that *S. cerevisiae* responds to phenolic-rich environment with processes which include conversion of the phenolic compounds, and that conversion could therefore be a possible mechanism for the cells to achieve tolerance to inhibitory compounds. In the present study, we showed that: (1) phenolic compounds are converted by *S. cerevisiae* and cell growth is not arrested during the conversion; (2) the conversion process of phenolic compounds is a sequential process with several intermediates, and may lead to detoxification since the conversion products are less toxic than their starting compounds; (3) some parts of the conversion pathway and mechanisms employed by *S. cerevisiae* may be common for all the phenolic compounds under investigation; (4) depending on the nature of the phenolic compounds involved, the conversion process may be rapid or slow.

In *S. cerevisiae*, the conversion and detoxification processes for handling many toxic substances leads to arrest of cell growth. Toxic metabolites, have also been known to arrest the growth of *S. cerevisiae*, mainly because they inhibit specific cellular processes inside the cell [22, 23]. Inhibitors such as furfural which are present in lignocellulosic materials have also been known to arrest growth and prolong the lag phase during conversion, severely affecting the cells redox metabolism, with potential impact on key cellular functions [24] In the present study, we observed a different relationship between growth and conversion of toxic compounds in *S. cerevisiae*. Simultaneous growth and conversion of the three phenolic compounds; coniferyl aldehyde, ferulic acid and *p*-coumaric acid was demonstrated in *S. cerevisiae*, even though the conversion was a detoxification process. Ahough previous studies have shown that coniferyl aldehyde causes a prolongation of the lag phase [19], the lack of lag phase prolongation may follow from the reduction of the concentration of coniferyl aldehyde from 1.4 to 1.1 mM during the scaling up of the process from the Bioscreen and Erlenmeyer flasks to the bioreactor, which, in combination with better aeration, agitation, and pH control in the bioreactor, may have favored yeast growth. The effect of the scale up to a bioreactor is also evident in the observation that the concentrations of compounds which resulted in a 80 % reduction in specific growth rate compared to the control in the Bioscreen-based screening, did not have the same level of inhibition in the bioreactor cultivation.

**Fig. 6:**
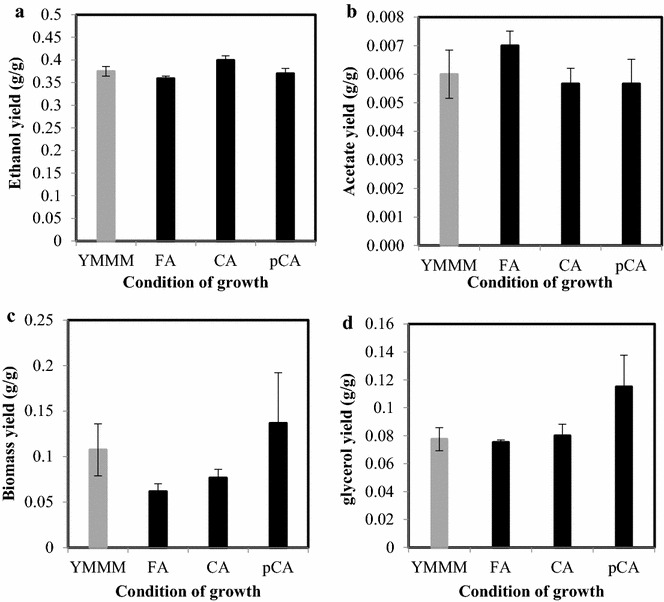
Yields of: **a** ethanol, **b** acetate, **c** biomass and **d** glycerol on glucose at 14 h. n = 7 P < 0.05. *YMMM* yeast minimal mineral medium, *CA* coniferyl aldehyde, *FA* ferulic acid, *pCA* *p*-coumaric acid

The most striking physiological differences between the inhibitor-containing cultivations and the -control were that the conversion of coniferyl aldehyde and that of ferulic acid similarly led to reduced biomass yields on glucose in cultivations containing any of these two phenolic compounds; that increased glycerol accumulation was found in cultivations containing *p*-coumaric acid; and that ethanol yields are not reduced in the presence of any of these three phenolic compounds. Also, the conversion of coniferyl aldehyde as well as that of ferulic acid did not lead to a reduced maximum specific growth rate for the cells (Fig. 2). Coniferyl aldehyde may have favored an increased ethanol yield (Fig. 6a), however we do not yet fully understand the relationship—if any—between the increased ethanol yield with sub-lethal concentrations of coniferyl aldehyde observed in this study. Although we have not investigated molecular mechanism responsible for the increased ethanol yield and reduced biomass yield in the presence of coniferyl aldehyde, the phenomenon has also been observed in yeast under stressful cultivation conditions in some other instances, examples of which are a *Saccharomyces cerevisiae* strain with mutated *GPD1* which has been engineered for reduced glycerol production [25], another case was in a cultivation of *S. cerevisiae* under aliphatic acid stress [4].

The significant reduction in maximum specific growth rate observed in cultivations containing *p*-coumaric acid may suggest ATP usage when converting *p*-coumaric acid into its less toxic products. We speculate that certain ATP-dependent reactions are involved in the conversion of *p*-coumaric acid. The reduction in biomass formation and increased glycerol production in cultivations containing *p*-coumaric acid may be indicative of a difference between the mechanism employed by the cell to detoxify *p*-coumaric acid and that employed for coniferyl aldehyde and ferulic acid. Another interpretation could be that the compounds have different cellular targets and modes of inhibition in the cells. We speculate that this difference would aid interpretation of the results of our previous study, which showed that coniferyl aldehyde, ferulic acid, and p-coumaric acid, together with 10 other phenolic compounds, have different effects on *S. cerevisiae* growth, and, based on the different effects, belong to different clusters of phenolic compounds [19].

**Fig. 7:**
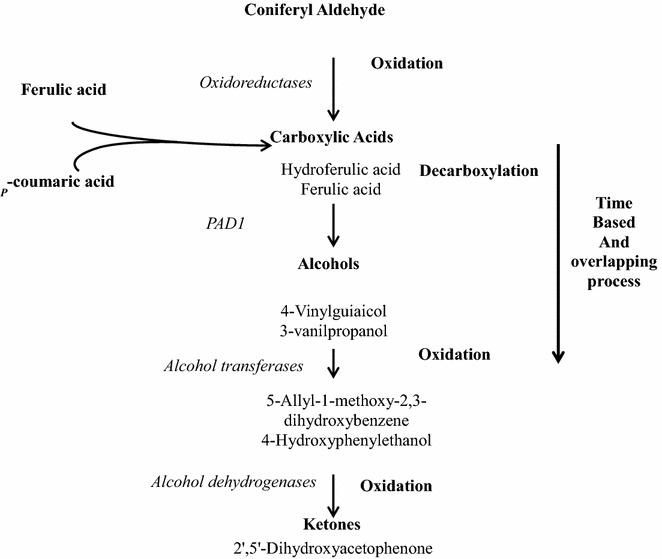
Proposed scheme for the conversion of phenolic compounds in *S. cerevisiae*

The results from this study enable us to hypothesize a conversion pathway that may be common for coniferyl aldehyde, ferulic acid, and *p*-coumaric acid, to further understand how *S. cerevisiae*, convert some phenolic compounds such as ferulic acid earlier reported [18, 21]. The trend observed in the conversion process followed a transition from phenolic aldehyde to phenolic acid, after which phenolic alcohols and ketones were formed. Similarly, in the case of ferulic acid, an isomer of ferulic acid was formed, as well as dihydroferulic acid, before other compounds were formed. In the case of *p*-coumaric acid, there was a conversion directly to alcohols. This observed conversion trend, coupled with the commonality of conversion products among the three phenolic compounds studied, despite their structural differences, is indicative of a common conversion pathway for phenolic compounds in yeast. Different conversion intermediates were formed during the individual conversion of the three different phenolic compounds (Table 2) but they nevertheless lead to similar or the same conversion end products. Based on the conversion data, it is evident that the point at which the conversion begins is dependent on the toxicity and structural complexity of the starting phenolic compound. In general, we therefore hypothesize that the conversion pathway may hold true for other phenolic compounds in the sequence we have observed, with a phenolic aldehyde first being converted to one or more phenolic acids, and the phenolic acids then being converted to phenolic alcohols. Phenolic acids initially may be converted to other phenolic acids, but, invariably, all are converted to phenolic alcohols and other categories of phenolics, as illustrated in the simplified conversion scheme in Fig. 7. The conversion of coniferyl aldehyde to ferulic acid may require the activity of a coniferyl aldehyde dehydrogenase enzyme which is well known in bacteria species such as *Pseudomonas*, but has not been identified in *S. cerevisiae*. For the conversion we have observed under aerobic cultivation condition, we hypothesize that an oxidoreductase is responsible for the conversion of coniferyl aldehyde that we have studied, this would be further investigated in subsequent studies. It has been shown that the conversion of ferulic acid in *S. cerevisiae* is facilitated by decarboxylases [17, 20], the most popularly known being phenyl acrylic acid decarboxylase. In addition, we hypothesize also that alcohol acetyl transferases and alcohol dehydrogenases play active roles in the conversion of further conversion of phenolic alcohols to phenolic ketones. These hypothesis shall be investigated in our subsequent studies.

Another interesting observation is the isomerization of ferulic acid. While isomerization of phenolic compounds had previously been proposed in *S. cerevisiae* [21], to the best of our knowledge, this is the first time the formation of a ferulic acid isomer has been observed. The specific enzymes involved, and the benefit gained by forming isomeric intermediates are currently not clear. Among the three phenolic compounds tested, the conversion of coniferyl aldehyde—which is the most toxic compound—was observed to be the most rapid. Within the first 48 h, coniferyl aldehyde was completely converted into its intermediate products, while the conversion of ferulic and *p*-coumaric acids lasted for 72 h. To survive in a toxic phenolic environment, yeast cells undertake a detoxification process that converts toxic phenolic compounds to less toxic derivatives through the formation of several intermediates, until significantly less toxic compounds are formed.

The ability of the *S. cerevisiae* to convert, detoxify the phenolic compounds and produce high ethanol yields that is comparable to the control is an interesting observation because the *S. cerevisiae* strain used in this study is an industrial strain. It may be indicative of the relevance of the strain for second generation bioethanol production using substrates rich in phenolic compounds inhibitors.

## Conclusion

We conclude that when *S. cerevisiae* is subjected to stress in a phenolics-rich substrate, *S. cerevisiae* responds by detoxifying its environment through the conversion of the toxic phenolic compounds, using a series of decarboxylation and oxidation processes into less toxic derivatives which the cells can then effectively cope with. This work highlights the in situ detoxification mechanisms in *S. cerevisiae* that can be exploited in developing phenolics resistant *S. cerevisiae* strains. Also, the close monitoring of the conversion process of coniferyl aldehyde, ferulic acid and *p*-coumaric acid as carried out in this study sheds light on the different stages of conversion and numerous intermediates formed in the process of detoxification of the phenolic compounds. Although the detailed metabolic pathway involved in this conversion process remains to be elucidated, the conversion explained in this study gives insight into the possibility of making high value phenolic compounds using *S. cerevisiae* as the cell factory. Although this is a single substrate study, through this work, we can however deduce that phenolic rich substrates such as pulping streams could be used for generating other products such as some of the phenolic conversion products which are useful for cosmetic, food and pharmaceutical applications. This therefore present an alternative use to lignocellulosic substrate other than production of biofuels.

## Methods

### Yeast strain

The industrial yeast strain *S. cerevisiae* Ethanol Red^®^ (Fermentis, a division of S. I. Lesaffre, Lille, France) was used for this study.

### Chemicals

All chemicals used in the preparation of the cultivation medium, including the phenolic compounds coniferyl aldehyde, ferulic acid, and *p*-coumaric acid, were purchased from Sigma-Aldrich GmbH, Germany.

All chemicals used in the chemical analyses of the starting phenolic compounds and their conversion products were of PA grade. Ethyl acetate, dichloromethane and acetone were purchased from Merck, Germany. 2,6-diethylnaphtalene and *N*,*O*-bis(trimethylsilyl)trifluoroacetamide (BSTFA) were purchased from Sigma-Aldrich, Germany. *O*-Vanillin was purchased from Fluka, Sweden.

### Medium preparation

The basal medium for the main cultivation was yeast minimal mineral medium (YMMM) [26]. Four cultivation media were used, (1) a control experiment without phenolic compounds in YMMM, (2) YMMM + 1.1 mM coniferyl aldehyde, (3) YMMM + 1.8 mM ferulic acid, and (4) YMMM + 9.7 mM *p*-coumaric acid. The concentration of phenolic compounds to be used in each medium had previously been determined by a toxicity experiment which has been reported previously [19].

### Cultivation

Each cultivation condition was performed in triplicate. The inoculum was cultivated in Erlenmeyer flasks incubated at 30 °C and 200 rpm for a period of 18 h in YMMM. A volume of inoculum that resulted in an OD_600_ of 0.2 was added to the main cultivation. The main cultivations were carried out in DASGIP parallel bioreactor systems comprising of two units, each holding four SR0700ODLS vessels (DASGIP, Jülich, Germany). The culture volume was 700 ml and the fermentors were preconditioned overnight at pH 5. Aeration was set to 1 vvm at an impeller speed at 400 rpm. The cultivations were run for 96 h and air aeration was maintained at a flow of 11.7 l/h throughout the cultivation. A feedback loop was created between the impeller speed and the dissolved oxygen probe signal to maintain aeration above 40 % of oxygen saturation.

Cultivation of yeast was done separately in the presence of each phenolic compound.

### Toxicity screening of phenolic compounds and conversion products on *Saccharomyces cerevisiae*

Experimental determination of the toxicity of the phenolic compounds and their conversion products was carried out by high-throughput toxicity screening using Bioscreen C MBR (Oy Growth Curves Ab Ltd, Finland), the set up was as we have described previously [19]. *S. cerevisiae* cultivations were done with different concentrations of single phenolic compounds in parallel. Growth was monitored in each cultivation and the concentration at which growth is not observed is noted. The toxicity limit for each phenolic compound is the concentration of a phenolic compound at which growth of the yeast is last observed. We have previously observed at this toxicity limit that the maximum specific growth rates and the final OD has been reduced to 80 % of the control, the elongation of lag phase is also 80 % more than that of the control.

### OD measurement of culture

Growth was followed by OD_600_ measurements using a Thermo Scientific GENESYS 20 Visible Spectrophotometer for measurement of the optical densities of cultures.

### Determination of dry cell weight

Determination of dry cell weight was performed in triplicate. 5 ml of culture was filtered using pre-dried and weighed filter paper discs of 0.45 μm pore size (Sartorius Stedim Biotech, Goettingen, Germany) on a water tap vacuum filter unit (Sartorius Stedim Biotech, Goettingen, Germany). The filter paper discs were dried in a microwave at 120 W for 15 min, weighed again and the biomass was determined from the difference.

### Determination of specific growth rates

Maximum specific growth rates was calculated from the plot of the natural logarithm of the measured optical density of the cultivation against the time of the cultivations. For cultivations in Bioscreen, the readings obtained from the Bioscreen were calculated back to standard spectrophotometric measurements at 600 nm via the formula:

1 $$\mathop {OD}\nolimits_{Spectro} = \frac{{\mathop {OD}\nolimits_{Bioscreen} }}{{PathLength\;({\text{cm}}) \times 1.32}}$$

where OD_spectro_ = equivalent OD on spectrophotometer at 600 nm, OD_Bioscreen_ = measured OD on the bioscreen

2 $$PathLength = \frac{{volume \;({\text{ml}})}}{{r^{2} \times \pi }}$$

where volume = culture volume in a well in the bioscreen plate; r = radius of the well.

Non-linearity at higher cell densities was corrected as described by Warringer et al. [27] using the formula:

3 $$OD_{cor} = OD_{obs} + (OD_{obs}^{2} \times 0.449) + (OD_{obs}^{3} \times 0.191)$$

where OD_cor_ = the corrected OD and OD_obs_ = the observed OD values, from which the average blank has been subtracted

### Determination of rates and yields

The specific consumption rate of the substrate (glucose) was determined using the formula

4 $$\mathop q\nolimits_{Substrate} = \frac{{\mathop \mu \nolimits_{{}} }}{{\mathop Y\nolimits_{(x/s)} }}$$

where q_*substrate*_ is the specific substrate consumption rate, µ the maximum specific growth rate, and Y_(x/s)_ the biomass yield coefficient.

The specific productivity rates of biomass, ethanol, acetate and glycerol were calculated using the formula:

5 $$\mathop {\mathop q\nolimits_{product} = \mathop q\nolimits_{Substrate} \times \mathop Y\nolimits_{(p/s)} }\nolimits_{{}}$$

where *q*_*product*_ is the specific productivity rate, q_*substrate*_ the specific substrate consumption rate, and Y_(p/s)_ the product yield coefficient.

During the respiratory growth phase, the biomass yield Y_(x/s)_, was calculated using a combination of glycerol, acetate and ethanol as substrate.

The yields of ethanol, glycerol, acetate and biomass from the consumed glucose were calculated during the exponential growth phase by plotting each of the products against the total consumed glucose. The yield for each product was obtained as the slope of a linear regression fitted to the plot. Average values of biological replicates were used as the final yield for each culture condition.

### Analysis of metabolites

Analysis of metabolites from the cultivation was performed by high performance liquid chromatography (HPLC) using a Dionex Ultimate 3000 HPLC unit (Thermo Scientific, Dionex Corporation, Sunnyvale, USA) equipped with an Aminex HPX-87H (Biorad, USA) column (300 mm × 7.8 mm), packed with 9 µm particles. The column temperature was set to 45 °C, and 5 mM H_2_SO_4_ was used as the mobile phase at a flow rate of 0.6 ml/min. A Shodex RI-101 RI detector and a Ultimate 3000 VWD 3100 variable wavelength ultraviolet detector coupled to the HPLC unit were used to quantify the metabolites.

### Time-based monitoring of the conversion of phenolic compounds and product formation

Simultaneously with the OD_600_ measurement, a 5 ml sample of culture was rapidly taken into 15 ml sample tubes and centrifuged at 0 °C and 5100 rpm for 5 min. Supernatants were kept frozen at −20 °C until qualitative analysis was carried out with gas chromatography–mass spectrometry (GC–MS).

Prior to GC–MS analysis, 0.5 ml of sample was mixed with 0.5 ml methyl acetate and 50 µl internal standard (100 µg/ml *o*-vanillin in ethyl acetate) and shaken. 0.45 ml of the mixture was dried using nitrogen until all the liquid had evaporated. 50 µl *N*,*O*-bis(trimethylsilyl)trifluoroacetamide (BSTFA) was then added, and allowed to react with the solid residue for 30 min at 80 °C. Finally, 950 µl dichloromethane and 50 µl external standard solution (111 µg/ml 2,6-diethylnaphtalene in acetone) was added.

The GC–MS analysis was performed using an Agilent HP7890A gas chromatograph (Agilent, Sweden) coupled with a Waters AutoSpec Premier magnetic sector mass spectrometer (Waters, UK). 1 µl of each sample was injected in splitless mode, and the injector temperature was held at 280 °C. Separation was carried out on a BPX5 capillary column (SGE Analytical Science, Sweden) of length 30 m, inner diameter 0.25 mm and film thickness 0.25 µm. Nitrogen with a flow of 1 ml/min was used as mobile phase. The temperature program was: 50 °C for 1 min, 10 °C/min to 300 °C, and then 300 °C for 10 min.

In the mass spectrometer, electron impact (EI+) was used for ionization. Mass spectra were recorded from *m*/*z* 40–400 with a total cycle time of 0.7 s. The resolution was 1000. Identification of the compounds with the highest abundance was performed by comparison of mass spectra with a NIST MS Search 2.0 library. The internal and external standards were used to determine tentative concentrations of the identified compounds.

### Statistical validation of data

All experimental data obtained in the course of the experiment were subjected to the student t test to determine if there was a significance level of difference with respect to the control. The number of replicates varied from 3 to 7, depending on the experiment. Therefore, a t test for two-sample assuming unequal variances was performed, with a significance level of probability set at p < 0.05. All error bars are standard deviations from the averages of multiple measurements of each parameter, all derived from biological replicates.
